# An Integrated Molecular Networking and Docking Approach to Characterize the Metabolome of *Helichrysum splendidum* and Its Pharmaceutical Potentials

**DOI:** 10.3390/metabo13101104

**Published:** 2023-10-23

**Authors:** Motseoa Mariam Lephatsi, Mpho Susan Choene, Abidemi Paul Kappo, Ntakadzeni Edwin Madala, Fidele Tugizimana

**Affiliations:** 1 Department of Biochemistry, University of Johannesburg, Auckland Park, Johannesburg 2006, South Africa; 201311232@student.uj.ac.za (M.M.L.); mchoene@uj.ac.za (M.S.C.); akappo@uj.ac.za (A.P.K.); 2 Department of Biochemistry and Microbiology, University of Venda, Thohoyandou 0950, South Africa; ntakadzeni.madala@univen.ac.za; 3 International Research and Development Division, Omnia Group, Ltd., Bryanston, Johannesburg 2021, South Africa

**Keywords:** natural products, anti-cancer, *Helichrysum splendidum*, computational metabolomics, molecular networking, molecular docking

## Abstract

South Africa is rich in diverse medicinal plants, and it is reported to have over 35% of the global *Helichrysum* species, many of which are utilized in traditional medicine. Various phytochemical studies have offered valuable insights into the chemistry of *Helichrysum* plants, hinting at bioactive components that define the medicinal properties of the plant. However, there are still knowledge gaps regarding the size and diversity of the *Helichrysum* chemical space. As such, continuous efforts are needed to comprehensively characterize the phytochemistry of *Helichrysum*, which will subsequently contribute to the discovery and exploration of *Helichrysum*-derived natural products for drug discovery. Thus, reported herein is a computational metabolomics work to comprehensively characterize the metabolic landscape of the medicinal herb *Helichrysum splendidum*, which is less studied. Metabolites were methanol-extracted and analyzed on a liquid chromatography–tandem mass spectrometry (LC-MS/MS) system. Spectral data were mined using molecular networking (MN) strategies. The results revealed that the metabolic map of *H. splendidum* is chemically diverse, with chemical superclasses that include organic polymers, benzenoids, lipid and lipid-like molecules, alkaloids, and derivatives, phenylpropanoids and polyketides. These results point to a vastly rich chemistry with potential bioactivities, and the latter was demonstrated through computationally assessing the binding of selected metabolites with CDK-2 and CCNB1 anti-cancer targets. Molecular docking results showed that flavonoids (luteolin, dihydroquercetin, and isorhamnetin) and terpenoids (tiliroside and silybin) interact strongly with the CDK-2 and CCNB1 targets. Thus, this work suggests that these flavonoid and terpenoid compounds from *H. splendidum* are potentially anti-cancer agents through their ability to interact with these proteins involved in cancer pathways and progression. As such, these actionable insights are a necessary step for further exploration and translational studies for *H. splendidum*-derived compounds for drug discovery.

## 1. Introduction

The genus *Helichrysum*, also known as “*impepho*” in isiXhosa (a South African native language) and “everlastings” in English, consists of around 600 species, the majority of which are found in South Africa [1]. *Helichrysum* species have been utilized in folklore medicine for at least 2000 years worldwide for the cure of several ailments such as gastric ulcers and gastritis, stomach damage, acute hepatitis, fever, edema, diuretic effects, and allergies [2,3,4,5]. Various *Helichrysum* spp. have also been reported to exhibit bioactive compounds that demonstrate a range of beneficial properties, including anti-inflammatory, anti-HIV, antioxidant, antibiotic, anti-cancer, and antiviral activities [6,7,8,9]. Several studies have investigated the phytochemistry of *Helichrysum* species, and most of these reports have focused on phenolic compounds. These studies examined a wide range of phenolic acids and flavonoids, which include both of free and glycosylated forms [10,11,12]. Both traditional medicine claims and the growing literature indicate that *Helichrysum* plants are a rich natural source of potential nutraceutical, pharmaceutical, and cosmetic candidates. Despite this growing attention to the phytochemistry of *Helichrysum*, there are still grey areas that hamper the full exploration of the plant. For instance, to date, the exploration of *Helichrysum* species has been predominantly limited to a few species such as *H. italicum*, *H. arenarium*, and *H. stoechas*; mostly, there is limited characterization of the metabolome of the plants. Furthermore, phytochemical characterization of other *Helichrysum* species, and a comprehensive interrogation of the *Helichrysum* metabolism, could illuminate the “dark matter” in the chemistry of the plant and its nutraceutical, pharmaceutical, and cosmetic potentials. Thus, reported herein is a computational metabolomics work to chart the chemical space of the less-studied *Helichrysum* species, *H. splendidum*, and computationally assess its anti-cancer potentials.

Over the last decade, metabolomics has been widely employed to assess the pharmacological efficacy and molecular processes of traditional herbal remedies [13,14]. Furthermore, recently introduced computational metabolome mining strategies have been impactfully driving the chemical and biological interpretation of untargeted metabolomics data, extracting functional information from spectral data. A recent study by Jan et al. (2022) [15] used computational metabolomics to identify the specific metabolites associated with the antioxidant and antidiabetic activities of four distinct varieties of *Morus alba* found in Kashmir, namely *Zagtul*, *Chtattatual*, *Chattatual Zaingir*, and *Brentul Kashmir*. Similarly, García-Pérez et al. (2021) [16] employed ultra-high-pressure liquid chromatography coupled to a quadrupole-time-of-flight mass spectrometer (UHPLC-QTOF/MS) to determine the phenolic composition of three *Bryophyllum* species: *Bryophyllum daigremontianum*, *Bryophyllum* × *houghtonii*, and *Bryophyllum tubiflorum*. The analysis revealed a total of 485 putatively annotated compounds, with flavonoids emerging as the most abundant subfamily of phenolic compounds. The presence of these phenolic compounds has been linked to the antioxidant, cytotoxic, anti-inflammatory, and antimicrobial activities observed in *Bryophyllum* plants. Phenolics such as flavonoids and terpenoids have been successfully isolated and purified from numerous plants such as *Salvia apiana*, *Tagetes lucida*, *Cussonia vantsilana*, *Helichrysum gymnocephalum*, and many others [17,18,19]. The purification of these abundant metabolites has allowed for further investigation into their potential health benefits and medicinal properties. Additionally, the development of pharmaceutical and nutraceutical products derived from these phenolics has led to new avenues for the treatment and prevention of various diseases. 

These accounts reflect that (medicinal) plants still represent a vastly rich resource that can be further explored for potential drug leads, and this is the case for *H. splendidum* reported in this study. However, traditional approaches in natural product (NP)-based drug discovery processes present discouraging challenges. These include rediscovery of known compounds, access to limited chemical space (of a medicinal plant) due to the lack of a comprehensive metabolomic landscape of the plant, and very time-consuming processes. As echoed above, with the rapid development of *omics* sciences, advancements in analytical instrumentation, and artificial intelligence technologies, efficient approaches are being developed to facilitate and improve NP-based drug discovery. Computational metabolomics, involving tandem mass spectrometry (MS/MS)-based molecular networking, combined with computational methods such as network pharmacology and molecular docking, represent time- and cost-effective approaches to investigate potential compounds (drug leads) prior to in vitro bioassays or chemical modifications for the (overall) accelerated drug discovery process [20,21,22]. Thus, contributing to these efforts, this study is a computational metabolomics work designed to comprehensively characterize the metabolome of *H. splendidum* (a less-studied *Helichrysum* plant); to our knowledge, this is the first report on the metabolomic map of this plant. Furthermore, this study intends to computationally assess and predict the drug-likeness of chemical classes in the *H. splendidum* metabolome, the molecular interaction between the proteins CDK-2 (cyclin-dependent kinase 2) and CCNB1 (cyclin B1) (involved in in the development and progression of cancer), and the inhibitors (ligands) identified in the plant’s extracts. CDK-2 and CCNB1 are two key molecules involved in cell cycle regulation, and they have been explored as potential anti-cancer targets due to their critical roles in controlling cell division. Targeting these molecules can disrupt the uncontrolled proliferation of cancer cells, making them attractive candidates for cancer therapy [23,24].

## 2. Materials and Methods

### 2.1. Chemicals and Plant Materials

As reflected above, this study was designed as an integration of LC-MS/MS-based molecular networking and molecular docking for a comprehensive characterization and prediction of the metabolome of a medicinal plant, *H. splendidum*, one of the less-studied *Helichrysum* plants. All chemicals used in this study were of pure grade quality and were acquired from various manufacturers. The organic solvent, methanol, was LC-MS-grade quality and was obtained from Romil (Cambridge, UK). Water was purified using a milli-Q gradient A10 systems Siemens (Munich, Germany). Formic acid was purchased from Sigma Aldrich (Munich, Germany).

The *H. splendidum* seeds were purchased from Seeds for Africa (https://www.seedsforafrica.co.za, accessed on 17 March 2023) and were planted in 4 L pots filled with potting soil mixed with Vita-Veg organic fertilizer (Talborne Organics, Bronkhorstspruit, South Africa). Eight pots were used, with three plants in each pot, and were placed under natural light. The plants were harvested at a 4-month growth stage. The stems and leaves of *Helichrysum splendidum* were freeze-dried, crushed, and the powdered samples were stored in a dried form at room temperature pending metabolite extractions.

### 2.2. Metabolite Extraction

One gram (1 g) of the powdered plant material was weighed and subjected to extraction in 20 mL of 80% aqueous methanol. Subsequently, the crude extracts underwent centrifugation at 2000 rpm for 30 min at 4 °C, followed by filtration through a 0.22 µm nylon filter into pre-labeled glass vials equipped with 500 µL inserts. The filtered samples were then stored at 4 °C until further analysis. To ensure experimental reproducibility, a total of twenty-four independent biological replicates were prepared, and three instrumental technical replicates were analyzed. In addition, quality control (QC) samples (pooled samples) were also prepared to assess the performance of the analytical platform and the quality of data generated and to correct any systematic errors.

### 2.3. LC-MS/MS Analysis

The prepared *Helichrysum splendidum* extracts were analyzed on a liquid chromatography–quadrupole time-of-flight mass spectrometry instrument (LCMS-9030 qTOF, Shimadzu Corporation, Japan) using a Shim-pack Velox C18 column (100 mm × 2.1 mm, 2.7 µm) (Shimadzu Corporation, Kyoto, Japan) at 55 °C. An injection volume of three µL was used, and a binary solvent system was utilized, comprising solvent A (0.1% formic acid in Milli-Q water) and solvent B (methanol with 0.1% formic acid). The chromatographic separation of analytes was carried out with a constant flow rate of 0.4 mL/min. A gradient lasting 53 min was employed with the following separation conditions: 10% B maintained for 3 min, a gradual increase from 10% to 60% B over 3 to 40 min, maintaining 60% B from 40 to 43 min, followed by a change to 90% B between 43 and 45 min and holding 90% B for 3 min. The gradient was then returned to initial conditions between 48 and 50 min, followed by a 3 min column equilibration time. The chromatographic effluents were subsequently subjected to analysis using a qTOF high-definition mass spectrometer (MS) operating in negative electrospray ionization (ESI) mode, based on preliminary optimizations. The MS instrument parameters were set as follows: interface voltage of 4.0 kV, interface temperature of 300 °C, nebulization and dry gas flow of 3 L/min, heat block temperature of 400 °C, DL temperature of 280 °C, detector voltage of 1.8 kV, and a flight tube temperature of 42 °C. Sodium iodide (NaI) was utilized as a calibration solution to ensure high mass accuracy. This solution contains NaI clusters with high masses and accounts for the calibration of higher *m*/*z* (Vékey, K., 1989). Both MS1 and MS2 (data-dependent acquisition, DDA) were simultaneously acquired for all ions with an *m*/*z* range between 100 and 1000 Da, surpassing an intensity threshold of 5000 counts. Fragmentation experiments were conducted using argon as a collision gas at a collision energy of 30 eV with a spread of 5 eV. 

### 2.4. Molecular Networking in the GNPS Analysis Environment 

The raw data obtained from the Shimadzu LCMS-9030 were converted to an open-source format (.mzML). The spectral data were then processed with MSDIAL, and the outputs were exported into the Global Natural Product Social (GNPS) (https://gnps.ucsd.edu, accessed on 17 March 2023) ecosystem for FBMN analysis [25,26]. The precursor ion mass tolerance was set to 0.05 Da, while the MS/MS fragment ion tolerance was set to 0.05 Da. Subsequently, a molecular network was constructed, and edges within the network were filtered to have a cosine score higher than 0.6 and a minimum of 4 matched peaks. Furthermore, edges connecting two nodes were retained in the network only if both nodes appeared in the respective top 10 of nodes most similar to each other. Additionally, the maximum size of a molecular family was limited to 100, and the lowest-scoring edges were eliminated from molecular families until the size of the family fell below this threshold. The spectra in the network were then searched against various GNPS spectral libraries including GNPS, SUPNAT, CHEBI, DRUGBANK, and FooDB. To retain matches between network spectra and library spectra, a score higher than 0.7 and a minimum of 6 peaks were required. The DEREPLICATOR tool was employed for the annotation of MS/MS spectra [27]. The Cytoscape software [28] was employed to visualize the molecular networks. Empirical formulae of all matched and some unmatched nodes were generated based on accurate mass and fragmentation patterns obtained from MS2 analysis. These formulae were then verified or tentatively annotated. Additionally, dereplication databases for natural products, such as KNApSAck [29], ChemSpider [30], PubChem [31], Dictionary of Natural Products [32], and available literature, were searched for further verification and annotation. 

In order to enrich chemical structural information within the generated molecular network, in silico structure annotations from GNPS Library Search and Network Annotation Propagation (NAP) were incorporated into the network using the GNPS MolNetEnhancer workflow (https://ccms-ucsd.github.io/GNPSDocumentation/molnetenhancer/ accessed on 17 March 2023). The consensus and fusion scores were calculated based on the top 10 candidate structures. Chemical class annotations were performed using the ClassyFire chemical ontology. Peptidic structural annotation was conducted using Dereplicator, while substructure annotation was performed using the MS2LDA interface in GNPS, including the Rhamnaceae, and GNPS Mass2Motifs in the search. Metabolite annotation was carried out at confidence level 2 of the Metabolomics Standards Initiative (MSI) [33].

### 2.5. Network Pharmacology

#### 2.5.1. Target Prediction, Data Acquisition, and Preprocessing

An unpublished Python script for target prediction was used, and the code for this script is available upon request. The complete curated Binding DB dataset, known as BindingDB_All.tsv, was downloaded from https://www.bindingdb.org/ (accessed on 23 May 2023). To ensure data consistency, any lines containing more than 283 tabs were removed. From each line, Uniprot IDs and counts were extracted. The dataset was then filtered to include only Homo sapiens, and a list of unique SMILES strings was generated after the filtering process. Utilizing the RDKit Python library [34], each unique SMILES string was converted into a molecule object and its corresponding Morgan fingerprint. Compounds that could not be converted were excluded from further analysis.

#### 2.5.2. Metabolite Similarity Analysis

Metabolite fingerprints were calculated, and the similarity between these fingerprints and the SMILES strings was evaluated using the Dice and Tanimoto similarity metrics, calculated with the RDKit Python library. A cutoff threshold of 0.6 was established to filter out pairs with low similarity scores. Additionally, a dictionary was created to map SMILES strings to their respective Uniprot IDs. For each key in this dictionary, the Uniprot IDs were consolidated into a single set, and a new dictionary was constructed to store the following information for each metabolite: SMILES strings with Dice and Tanimoto similarities, mean Dice and Tanimoto similarity scores, and predicted target Uniprot IDs based on the Dice and Tanimoto similarity metrics.

#### 2.5.3. Output Preparation and Analysis

An output DataFrame was generated, containing information on the metabolites, mean Dice similarity, mean Tanimoto similarity, and predicted targets based on the Dice and Tanimoto cutoffs. The overlap between the predicted targets was calculated and included in the output DataFrame. For each metabolite, the frequency of predicted targets based on Dice and Tanimoto similarities was determined. These frequency scores were normalized by dividing them by the total number of predicted targets and sorted in descending order. The ranked targets based on the Dice and Tanimoto similarity rank scores were added to the metabolite dictionary. The output DataFrame was augmented with mean Dice and Tanimoto similarity scores, as well as rank scores for each metabolite based on the cutoffs. To normalize the scores, they were divided by the target frequency. The normalized scores for the Tanimoto and Dice filters were then computed and appended to the output DataFrame.

#### 2.5.4. Compound–Target Network

To compute the compound–target network, cancer targets were retrieved from GeneCard human gene database [35], and the overlapping targets between the dataset’s retrieved targets and the predicted ones were then used to generate the compound–target network. The list of the overlapping targets was visualized in Cytoscape, and network graphs were generated for each compound and its respective targets to identify the most bioactive *H*. *splendidum* compounds. 

#### 2.5.5. Protein–Protein Interaction Network and Gene Ontology Enrichment Analysis

To gather protein–protein interaction (PPI) data, STRING [36] was utilized, set at a minimum interaction score of >0.7 and limited to Homo sapiens, narrowing the data to human-specific interactions. For visual representation of the network graphs, Cytoscape was used. Highly connected sub-networks within the PPI network were then generated by employing the Molecular Complex Detection (MCODE) plugin in Cytoscape. A gene ontology (GO) enrichment analysis network of the cancer targets was conducted on Metascape [37]. The gene identifiers of the targets in a list format were uploaded on the provided input field, and the organism of interest (*Homo sapiens*) was specified to ensure accurate enrichment analysis. Metascape then generated a comprehensive enrichment analysis report, including enriched GO terms, associated biological processes, molecular functions, cellular components, and pathway information. 

### 2.6. Molecular Docking

#### 2.6.1. Protein and Ligand Preparation

The crystal structures of human cyclin-dependent kinase 2 (CDK-2) (PDB ID: 2CCH) and cyclin B1 (CCNB1) (PDB ID: 2B9R) proteins were obtained in .pdb format from the Protein Data Bank (PDB) (https://www.rcsb.org/ accessed on 23 May 2023), a global repository used for accessing 3D structures of biological macromolecules [38]. The proteins were prepared using Discovery Studio software (version 20) to ensure their optimal structure and conformation for subsequent docking analysis. Preparation involved removal of water molecules or ligands that might interfere with the docking process. The initial 3D structures of the selected ligands were retrieved in .sdf format from PubChem (https://pubchem.ncbi.nlm.nih.gov/ accessed on 23 May 2023), a public information system for analyzing the bioactivity of small molecules [39], and were prepared using Open Babel [40]. Structure optimization was employed by applying force-field-based energy minimization algorithms to minimize steric clashes, correct bond lengths and angles, and improve the overall ligand geometry.

#### 2.6.2. Docking Method

Molecular docking was performed using Autodock Vina within the PyRx software environment [41]. To initiate the docking process, the prepared receptor structures (proteins) and ligand structures were imported into PyRx. During the docking simulation, Autodock Vina evaluated the binding energies of each docking pose based on a scoring function that considered various factors, including steric clashes, hydrogen bonding, and electrostatic interactions. The scoring function helped identify the most energetically favorable binding pose, which represented the predicted binding mode of the ligand within the receptor. The most favorable pose was then saved and visualized using Discovery studio. The visualization facilitated the identification of key interactions between the ligand and receptor, such as hydrogen bonds, hydrophobic interactions, or electrostatic interactions, which played a crucial role in determining the binding affinity and biological activity of the ligand. 

## 3. Results and Discussion

### 3.1. The Metabolomic Chart of H. splendidum Methanol Extracts 

**Figure 1:**
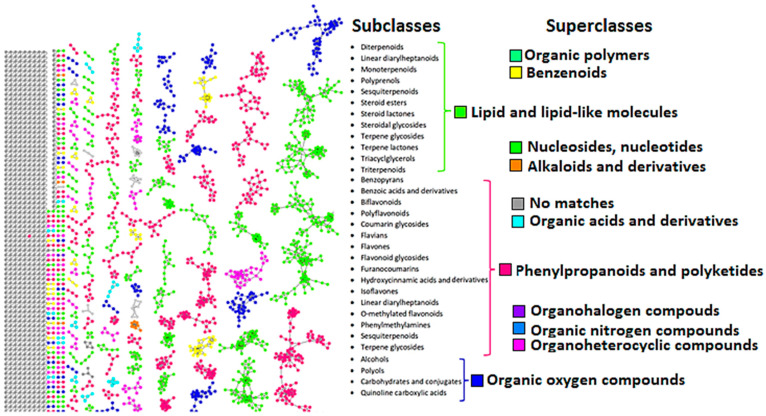
***Helichrysum splendidum* chemical space visualization with molecular networking**. MolNetEnhancer network analysis of spectral data from *H. splendidum* methanol extracts. The network shows three major metabolite classes (with different subclasses) identified, which define the *H. splendidum* chemical space: lipid and lipid-like molecules, phenylpropanoids, and organic oxygen-containing compounds. The colored nodes represent the MS/MS spectra matched to GNPS libraries, and unmatched nodes are represented in grey.

Chromatographically, the methanol extracts from the *H. splendidum* plant are highly complex mixtures of metabolites with a wide range of polarities (Supplementary Figure S1A). To further decode this chemical space, spectral data from the *H. splendidum* methanol extracts were mined and visualized using molecular networking strategies housed in the GNPS ecosystem (Section 2.4). The computed feature-based molecular network (FBMN) contained 5710 nodes (Supplementary Figure S2). Among the total nodes observed from the FBMN, 194 hits were matched to known metabolites present in the different databases (Section 2.4), and 59 of these metabolites were further validated through manual confirmation by comparing the mirror spectra, mass differences, and retention times (Table 1), to ensure the accuracy of metabolite identification to levels 2 and 3 as classified by the Metabolomics Standard Initiative (MSI). Furthermore, to explore the fragmentome and to predict molecular family and chemical class annotation, both MS2LDA and in silico annotation tools (NAP and DEREPLICATOR) were applied, respectively (Section 2.4). Integrating the outputs from FBMN, MS2LDA, and in silico tools in an enhanced molecular network, the MolNetEnhancer workflow provides the putative chemical structural information at the chemical superclass and subclass levels (Figure 1). MolNetEnhancer combines library matching, discovery of molecular substructures, in silico fragmentation tools, and chemical classification ontologies into a single molecular network [42]. By incorporating experimental and predictive outputs into multi-informative MN layers, MolNetEnhancer reveals molecular families, subfamilies, and structural nuances among family members, thereby facilitating a more comprehensive metabolite assignment at different molecular levels, ranging from broad chemical classes to diverse structural scaffolds and candidate structures [42]. As such, MolNetEnhancer provides a comprehensive overview of chemical space present in MS experiments. Thus, in this study, MolNetEnhancer offered the putative chemical classification of compounds identified in the *H. splendidum* extracts at the subclass level, such as benzenoids, organoheterocylic compounds, phenylpropanoids, organic oxygen compounds, lipids, organic acids, nucleosides, and alkaloids (Figure 1, Table 1).

Thus, our results indicate, for the first time, that the *H. splendidum* metabolomic chart is characterized by a wide spectrum of chemical (sub)classes, which could be grouped into superclasses ranging from lipid and lipid-like molecules to organic oxygen compounds (Figure 1, Table 1). The predominant chemical superclasses in the *H. splendidum* metabolomic landscape are (i) lipids and lipid-like molecules and (ii) phenylpropanoids and polyketides (Figure 1). Functionally, lipids serve a range of biological roles in plant cells, both structurally and as bioactive compounds. For instance, phospholipids and sphingolipids are cell membrane components that participate in cell signaling; galactolipids are chloroplast membrane components that participate in photosynthesis, and triacylglycerols (TAGs) are used for energy storage [43,44]. Furthermore, some of these specialized metabolites have been documented to provide specific health benefits to humans. For instance, depending on their modes of action, plant-derived lipids can stimulate the human immune system, decrease inflammation, enhance bone health, support eye and brain function, mitigate the risk of coronary heart disease, and exhibit antioxidant and anti-carcinogenic properties [45,46,47]. Phenylpropanoids and polyketides (Figure 1), on the other hand, are oxy-prenylated secondary metabolites that represent a unique group of natural products. In the past two decades, oxy-prenylated specialized metabolites have gained significant attention from researchers worldwide due to their noteworthy pharmacological activities, therapeutic potential, and beneficial impact on human health [48]. These phytochemicals have demonstrated in vitro and in vivo effects, making them promising candidates for the prevention and treatment of acute and chronic diseases. Extensive studies have unveiled the diverse interactions of oxy-prenylated secondary metabolites with various biological targets, leading to their recognized roles in anti-carcinogenesis, anti-inflammatory responses, neuroprotection, immune modulation, blood regulation, and metabolic regulation [49]. The richness of the two predominant chemical superclasses, i.e., lipids and lipid-like molecules and phenylpropanoids and polyketides in *H. splendidum* (Figure 1), may therefore account for the medicinal properties of this plant reported in folklore such as anti-inflammatory and anti-cancer activities [7,50]. 

### 3.2. Health Benefits from the H. splendidum Chemistry: The Case of Flavonoids and Terpenoids

**Figure 2:**
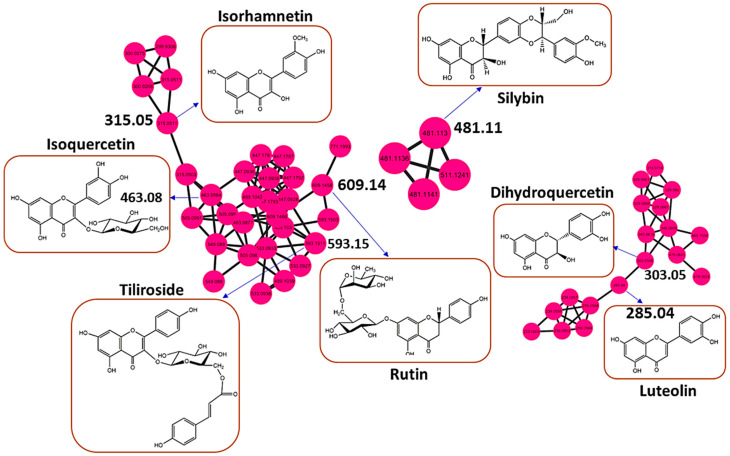
**Molecular network highlighting potentially bioactive flavonoids in *Helichrysum splendidum* extracts**. A cluster of flavonoids characterized by a molecular network showing different flavonoid metabolites including isorhamnetin, isoquercetin, silybin, dihydroquercetin, tiliroside, rutin, and luteolin.

As revealed in Section 3.1, the metabolic landscape of *H. splendidum* comprises a wide spectrum of phytochemicals, some of which arguably exhibit various biological activities. One of the chemical clusters of interest is the flavonoid family, which is in the superclass of phenylpropanoids and polyketides (Figure 1). The flavonoid family comprises compounds such as isoquercetin, isorhamnetin, tiliroside, silybin, rutin, luteolin, and dihydroquercetin (Figure 2, Supplementary Figure S1B). Based on the MN philosophy, this flavonoid cluster also contains unknown metabolites or ion features, which are structurally similar or related to these known flavonoid metabolites. Such extrapolation suggests that there could be more (novel) flavonoid-like molecules in *H. splendidum* methanolic extracts. Furthermore, various studies have suggested that *Helichrysum* species represent an abundant source of flavonoids, some of which possess activities such as antioxidant, anti-inflammatory, wound-healing, antimicrobial, photoprotective, and anti-carcinogenic [50,51,52,53]. Isoquercetin, isorhamnetin, tiliroside, silybin, rutin, luteolin, and dihydroquercetin have been previously reported to have inhibitory effects on different cancerous cell lines [54,55,56,57,58,59,60]. Due to their therapeutic properties, flavonoids derived from plants have been investigated for their potential use in cancer chemotherapy. Flavonoids have shown efficacy against various cancer types by impeding cell cycle progression, protecting cells against external damage, suppressing mutations, inhibiting prostaglandin synthesis, and preventing carcinogenesis in animal models [61]. Yagura et al. (2008) [62] reported on the presence of anti-carcinogenic compounds in *Helichrysum maracandicum* where naringenin chalcone exhibited a strong anti-proliferative activity against cultured cells of SENCAR (SENsitive to CARcinogenesis) mouse strain (model) in an in vitro assay. Thus, the presence of a wide range of flavonoid compounds in *H. splendidum* (Figure 1 and Figure 2) qualitatively suggests flavonoid-linked anti-cancer properties of the plant, which is worth investigating (Section 3.3). 

**Figure 3:**
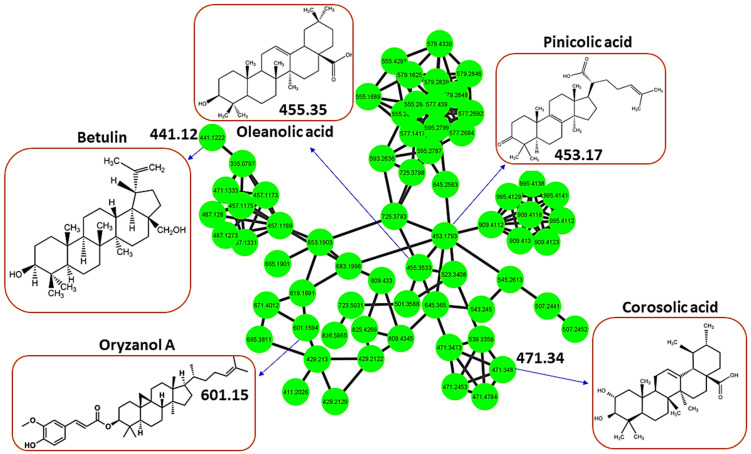
**Molecular network highlighting potentially bioactive terpenoids in *Helichrysum splendidum* extracts**. A cluster of terpenoids characterized by a molecular network showing different terpenoid metabolites, including oleanolic acid, pinicolic acid, betulin, oryzanol A, and corosolic acid.

In addition to profiled flavonoids (part of the phenylpropanoids and polyketides chemical superclass), the metabolome of *H. splendidum* is predominantly characterized by lipids and lipid-like molecules (Figure 1), of which terpenoids have been reported to possess anti-cancer activities. The *H. splendidum* terpenoid profile comprises betulin, oleanolic acid, oryzanol A, pinicolic acid, and corosolic acid metabolites (Figure 3). Terpenoids (oxygen-containing hydrocarbons) are a modified group of terpenes with diverse functional groups and rearranged or eliminated oxidized methyl groups at different positions. The classification of terpenoids is based on the number of carbon atoms they contain, ranging from mono-, di-, tri-, and sesqui- to sesterpenoids. The majority of terpenoids, which vary in their structural makeup, are physiologically active, and they are thought to be potentially effective in cancer pharmacotherapy due to their ability to produce a wide range of functional groups [63]. The structural features of terpenoids that confer anti-cancer properties can also vary depending on the specific compound and the target cancer cell type. Betulin (Figure 3) has been demonstrated to possess cytotoxic effects against numerous human neoplastic cell lines, including cervical (HeLa), liver (HepG2, SK-HEP-1), lung (A549), breast (MCF-7), melanoma (G361), colorectal carcinoma (HCT116, HT29), and prostate tumor (PC-3) cell lines [64,65]. An increasing body of evidence suggests that the anti-cancer activity of betulin is primarily mediated through apoptosis activation [66]; however, the precise molecular mechanisms underlying the anti-cancer action of betulin still remain to be investigated. Similarly, ursane-type terpenoids, such as corosolic acid and oleanane types, such as oleanolic acid (Figure 3), have been documented for their anti-proliferative activities against gastric (NCI-N87), colorectal (HCT15), cervical (HeLa), glioblastoma (U291, U373, and T98G), and colon (HT29) cancer cell lines [67,68]. The potential anti-cancer activity of *H. splendidum* can therefore be ascribed to the presence of the identified terpenoids in the methanol extracts such as oleanolic acid and corosolic acid (Figure 3) with reported anti-proliferative properties together with the structurally similar compounds as seen from the MN.

Most flavonoids and terpenoids, found in a wide range of edible and medicinal plants, have been suggested to possess chemo-preventive and cytotoxic effects against various types of cancers via diverse mechanisms. However, the clinical use of these compounds is still very limited and challenging due to various constraints and bottlenecks. These include decoding the chemical space in which these compounds are found and their isolation and purification from their natural resources; characterization and understanding the molecular mechanisms governing the chemo-preventive and cytotoxic effects of these compounds; the cost and time needed for epidemiological studies; and several pharmacokinetic challenges (e.g., bioavailability, drug–drug interactions, and metabolic stability). To address some of these challenges, numerous approaches are being devised and applied, such as the increasing development and exploration of computational and bioinformatics methods. These help to rapidly gain actionable insights into possible molecular mechanisms that define bioactivities of (these) metabolites, modeling interactions at the atomic level between the metabolites and predicted macromolecules [69]. Such emerging efforts, leveraging computational strategies such as network pharmacology and molecular docking, represent a paradigm shift in the drug discovery process and are time- and cost-effective approaches to determine potential (bioactive) compounds prior to in vitro bioassays or chemical modification, subsequently accelerating the process. Thus, the work reported herein contributes to these ongoing efforts, with a focus on *H. splendidum* chemistry and its potential anti-cancer activities.

### 3.3. Network Pharmacology and Molecular Docking of Flavonoids and Terpenoids from H. splendidum in the Binding Pocket of CDK-2 and CCNB1

Molecular docking is extensively employed to predict the mechanism of action and elucidate the structure–activity relationships of natural products. Docking aims to accurately determine the orientation of a ligand within a protein’s binding pocket and assess the strength of the binding using a docking score [70]. The 3D structure of the protein in question is obtained either from X-ray crystallography, NMR data, or generated through homology modeling. Ligand molecules are then computationally positioned within the binding pocket to analyze their potential interactions with the target, thereby identifying the crucial binding features of the molecule. This in silico method represents a valuable filtering tool in the quest for new bioactivities associated with natural products and can be used to find and uncover novel activities for previously characterized plant-derived natural products [69]. The potential of flavonoids and terpenoids to hinder cell proliferation and trigger apoptosis or autophagy in human cancer cells has recently sparked significant interest regarding their prospects as anti-cancer agents [71,72]. 

Several epidemiological studies substantiate the preventive properties of flavonoids in relation to cancer, and numerous studies have sought to establish correlations between the structural characteristics and anti-cancer activity of flavonoids. Despite extensive research on flavonoids and terpenoids and their potential anti-cancer properties, there is still limited understanding of how the structure of these compounds relates to their anti-cancer activity. This lack of knowledge can be attributed to incomplete information regarding the interactions between these compounds and their targets. As echoed above, to address this gap, functional sites of protein molecular surfaces and protein and ligand interactions can be computationally predicted [22,73]. These efforts, such as the study reported herein, hold the potential for uncovering novel therapeutic agents.

**Figure 4:**
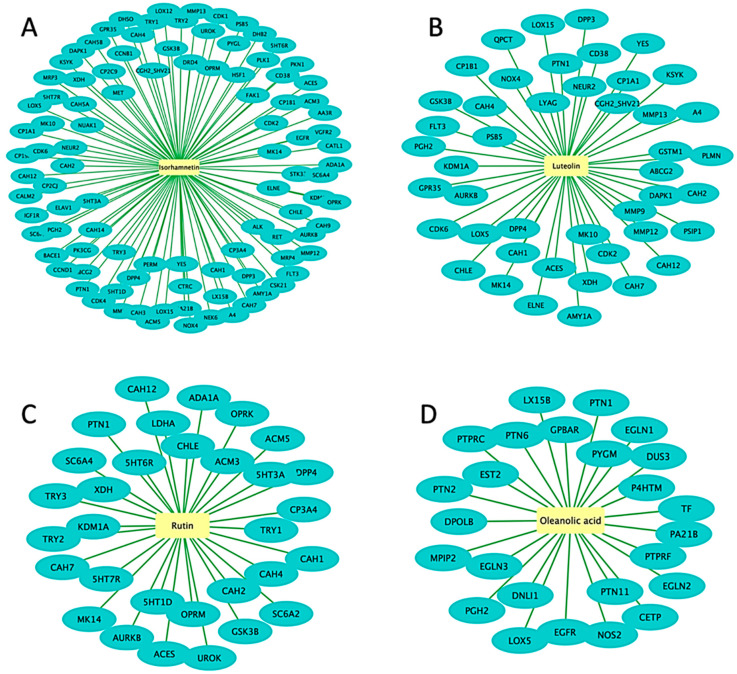
**Compound–target network predicting bioactive *H. splendidum* compounds**. The compound network predicts the interactions between the compounds isorhamnetin (**A**), luteolin (**B**), rutin (**C**), and oleanolic acid (**D**) and their target proteins involved in cancer biology.

Thus, in this study, to explore the potential molecular targets of flavonoids and terpenoids as promising anti-cancer agents, molecular docking was employed using various enzymes and receptor proteins involved in cancer pathways. Firstly, network pharmacology (NP), a drug discovery discipline that uses computational biological tools to elucidate drug interactions with multiple targets, was employed [74]. NP integrates systems’ biology and bioinformatic tools to decipher the complex relationship between drugs, potential targets, and diseases, thus providing a promising approach for disease action mechanisms and the identification of potential bioactive compounds [75]. In this study, network pharmacology was used to predict the bioactive flavonoids and terpenoids present in the metabolomic map of the *H. splendidum* (Figure 1, Figure 2 and Figure 3). Potential cancer targets were computed using protein–protein interaction (PPI) network construction and analysis and gene ontology (GO) and Kyoto Encyclopedia of Genes and Genomes (KEGG) pathway analysis. To identify the bioactive compounds in *H. splendidum*, which would be used for molecular docking, a compound–target network was generated (Figure 4). A compound–target network generates prediction interactions between chemical compounds and their targets such as proteins or receptors. Thus, Figure 4 reveals isorhamnetin, luteolin, rutin, and oleanolic acid as bioactive compounds due to the high number of interactions observed between these compounds and the different cancer target proteins predicted from relevant databases. In compound–target networking, a higher number of interactions (multiple targets) is correlated with (potentially) increased bioactivity [76]. These compounds were then chosen for the additional computational ligand–target interaction analysis.

**Figure 5:**
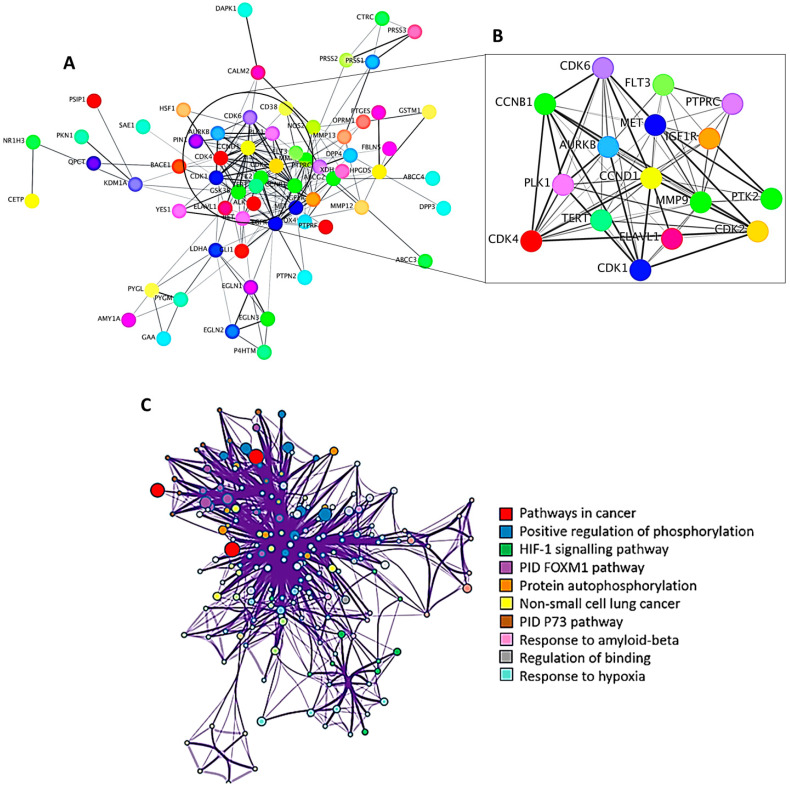
**Protein–protein interaction (PPI) and gene ontology (GO) enrichment analysis**. (**A**) PPI represents a network of protein–protein interactions, (**B**) highlighting distinct clusters of cancer targets using the MCODE algorithm, and (**C**) enrichment of KEGG pathways.

As mentioned above, PPI networking was used to predict specific protein targets that are implicated in cancer disease. A total of 65 predicted protein targets were imported into STRING to generate a PPI network (Figure 5A). PPI networks represent the physical and functional interactions between proteins in a biological system under specified physiological conditions [77]. These networks therefore provide a systems-level perspective by considering the interactions of target proteins rather than studying them in isolation. This leads to a deeper understanding of the biological mechanism of the disease in question and potential therapeutic applications. The MCODE plugin was then employed to identify the most densely connected regions within the PPI network, and the top 10 core cancer targets were CDK1, CDK2, CDK6, CCNB1, CDK4, CCND1, PLK1, AURKB, HIF1A, and GSK3B (Figure 5B). 

To further analyze and interpret the functional characteristics of the target protein candidates, GO enrichment analysis was employed (Figure 5C). During enrichment analysis, the input gene list (target proteins) is compared to numerous gene sets specified by their involvement in specific biological processes and pathways [37]. The top 10 pathways revealed by the GO enrichment analysis were then highlighted together with the network of enrichment terms (Figure 5C). The enrichment analysis showed the direct participation of the target proteins in the pathways known to be associated with cancer. The ten pathways emerged as the most significant, surpassing the rest of the pathways and biological processes with a *p*-value of 10–20 (Figure 5C). These findings therefore suggest a strong connection of the identified target proteins to cancer-related processes and shed light on the significance of these pathways functionally. Following the identification of bioactive compounds in *H. splendidum* using a compound–target network and target proteins through GO enrichment analysis (Figure 4 and Figure 5), exploration and investigation of metabolite–protein interactions, using molecular docking, were then employed.

**Figure 6:**
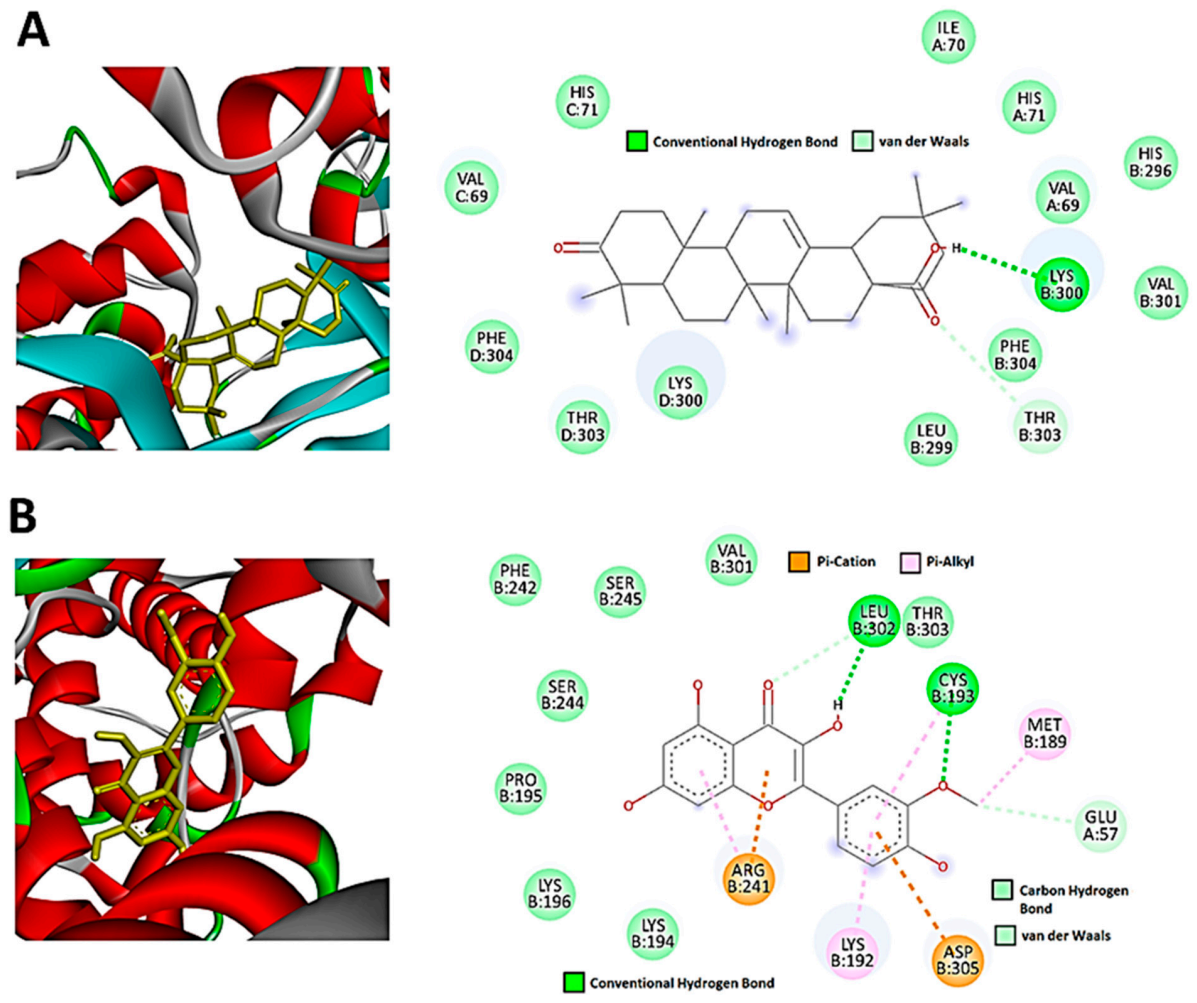
**Flavonoid and terpenoid molecular interactions with CDK2 protein residues**. Two-dimensional ligand-CDK2 diagrams of (**A**) oleanolic acid and (**B**) isorhamnetin with docking scores of −8.9 and −7.8, respectively, visualized using Discovery studios.

The molecular docking performed in this work was an attempt to forecast the likely modes of interactions and mechanisms of the identified bioactive compounds in the *H. splendidum* extract. Isorhamnetin, luteolin, rutin, and oleanolic acid (Figure 4) were docked into binding sites of CDK2 (representative of the other CDKs identified by the PPI network) and CCNB1 targets. Findings from the molecular docking study are presented in Supplementary Table S1. From these, oleanolic acid and isorhamnetin had the highest docking scores of −8.9 and −7.8. Figure 6 presents the interaction diagrams (3D and 2D) of the respective compounds and their targets. For instance, the interactions between the CDK-2 enzyme and oleanolic acid were characterized with the strongest interaction formed by hydrogen bonds with lysine (LYS300) and the amino acid fraction of the CDK-2 enzyme (Figure 6A). Van der Waals interactions were also formed between the two molecules. Isorhamnetin, on the other hand, in addition to hydrogen bonds formed by LYS302 and cysteine (CYS193), formed pi-cations and pi-alkyl interactions. Pi-cation interactions occur due to a positively charged amino acid residue of the protein and the aromatic system of the compound, as observed in Figure 6B. Pi-alkyl interactions were also observed in which the benzene ring of the compound and the alkyl side chain of the amino acid residues form an interaction when in close proximity to stabilize the binding of the molecules. Furthermore, the results reveal that hydroxyl groups (of both terpenoids and flavonoids) are crucial in the metabolite–target interactions (Figure 6). As such, the molecular basis of the bioactivities of *H. splendidum*-derived terpenoids and flavonoids were computationally revealed, particularly the banding to the CDK-2, an anti-cancer target protein. 

Thus, these results (Figure 6) reveal and demonstrate strong interactions between *H. splendidum*-derived flavonoids/terpenoids and the CDK-2 protein, which implies that these specialized metabolites are predictively able to alter CDK-2 structural conformations, subsequently halting or inhibiting their activities. Cyclin-dependent kinases (CDKs) are a group of twenty serine/threonine kinases that have essential roles in governing cell proliferation, transcription, differentiation, and metabolism [78]. CDK-2, a member of the CDK protein family, plays a crucial role in the transition from the G1 to S phase of the cell cycle and is typically overexpressed in human malignancies while having minimal expression in most normal tissues [79]. Interestingly, numerous studies have demonstrated that inhibiting CDK-2 can induce apoptosis in cancerous cells while causing minimal harm to normal cells [80,81,82]. From these observations, we can postulate that *H. splendidum* has pharmaceutical potential as a source of bioactive and druggable compounds, particularly from terpenoid and flavonoid structural classes (Figure 6), for cancer treatment. The chemical map of the plant shows richness in flavonoid content (Figure 2 and Table 1), and from these computational models (Figure 6), it is evident that these specialized metabolites could functionally inhibit cancer cell growth through their interactions with protein kinases. The latter have become critical pharmacological targets due to the development of numerous kinase inhibitors [83], and identifying molecular targets involved in cancer incidence has become a critical step in developing prospective anti-cancer agents [84,85]. 

**Figure 7:**
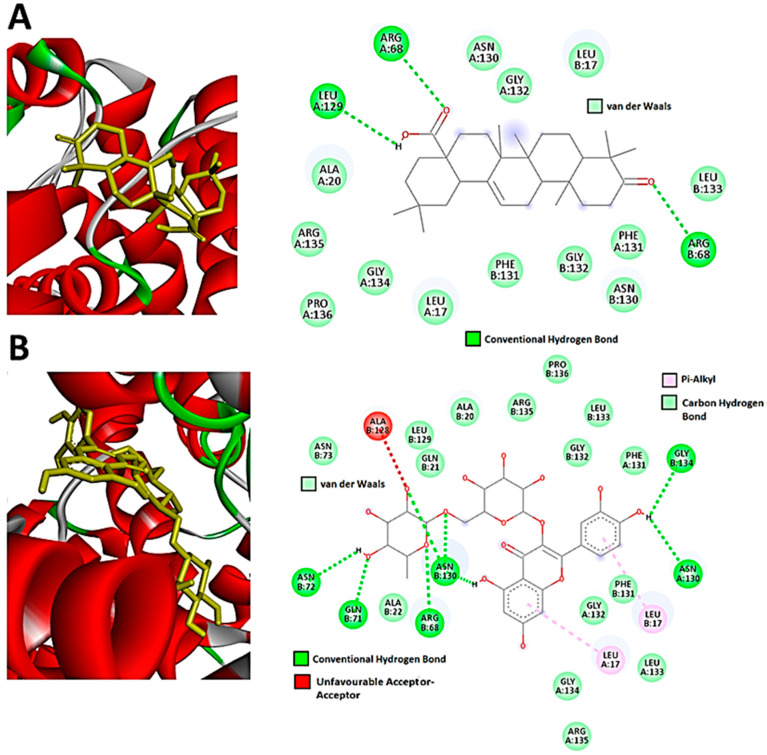
**Flavonoid and terpenoid molecular interactions with CDK2 protein residues.** Two-dimensional ligand-CDK2 diagrams of (**A**) oleanolic acid and (**B**) rutin with docking scores of −8.9, visualized using Discovery studios.

For CCNB1, oleanolic acid and rutin both had the highest docking scores of −8.9. These ligands also adopted a similar binding mode, in which the observed interactions were mainly hydroxyl and carboxyl groups of the compounds interacting with the different amino acid residues of CCNB1 and forming hydrogen bonds (Figure 7A). Computationally, it was observed that oleanolic acid forms hydrogen bond interactions with the amino residues (LEU129 and ARG68) of the CCNB1 enzyme. The high binding affinities observed can be attributed to the hydrogen bonds and van der Waals interactions. Rutin similarly formed hydrogen bond interactions with the CCNB1 target. The number of CCNB1 amino acid residues forming these interactions with the compound was higher (ASN72, GLN71, ARG68, ASN130, GLY134) as compared to oleanolic acid. Pi-alkyl interactions were also observed between LEU17 and the benzene groups of rutin (Figure 7B). As such, these results (Figure 7) computationally reveal and demonstrate strong interactions between *H. splendidum*-derived flavonoids/terpenoids and the CCNB1 protein, which implies that these specialized metabolites would alter the structural conformations of this protein and subsequently halt or inhibit its activities. Cyclin B1 (CCNB1), a key protein involved in the regulation of the cell cycle, plays a crucial role in cancer therapy. CCNB1 forms a complex with CDK1 to aid in the progression of cells through the G2/M phase transition in the cell cycle. This complex controls numerous processes which are required for cell division such as entry into mitosis and chromosomal segregation [86]. 

CCNB1 has emerged as a promising candidate for anti-cancer therapy due to its essential role in cell cycle control. Attempts have been made in the last decade to develop novel CCNB1 inhibitors in response to the observed overexpression of CCNB1 in cancer cells originating from various sources such as breast, colorectal, prostate, and hepatocellular cancers [87,88,89]. Recently, Aljohani et al. (2022) [90] reported that high CCNB1 protein expression was associated with aggressive tumor behavior resulting in large tumor formation in breast cancer. As a result, blocking CCNB1 signaling in both tumor endothelium and malignant cells has emerged as a prospective target for developing novel cancer therapies. In this same line of efforts, our study suggests that the *Helichrysum splendidum* metabolomic landscape may contain potential CCNB1 inhibitors, as seen from the molecular docking results. As previously mentioned, CCNB1 and CDKs form a complex in the cell cycle which in turn phosphorylates a multitude of downstream targets that are responsible for the progression of mitosis. Interestingly, the findings from our study highlighted that *H. splendidum* extracts can potentially inhibit both proteins (Figure 6 and Figure 7), suggesting a more aggressive disruption of the intricate network of the cell cycle, which could lead to accelerated cell cycle arrest and inhibition of tumor growth.

Thus, the computational modeling reveals that *H. splendidum*-derived flavonoids and terpenoids possess anti-cancer bioactivities through their molecular interactions with proteins involved in cancer progression. Such actionable insights pave the way for in vitro and in vivo confirmatory studies. Furthermore, extrapolating from the molecular networking principles, i.e., structurally similar compounds are grouped together in a network cluster, it can be postulated that structurally related metabolites, as revealed by a molecular network, could possess similar bioactivities. This can be illustrated by the case of oleanolic acid, which was found to have the highest binding score of -8.9 when docked into the binding sites of CDK2 and CCNB1. From the molecular network (Figure 3), pinicolic acid is structurally similar to oleanolic acid and could therefore possess similar anti-cancer bioactivity. Similarly, isorhamnetin and rutin were found to form clusters in Figure 2, suggesting an abundance of potential anti-cancer compounds within the *H. splendidum* extract. This points to a large pool of potential anti-cancer compounds present in the *H. splendidum* metabolomic map, and most of it is yet to be investigated. Thus, our study contributes to ongoing efforts to comprehensively characterize the (bio)chemistries of medicinal plants, particularly in South Africa. Combining molecular networking with molecular docking allowed for the exploration of a broader chemical space of *H. splendidum*, the less-studied *Helichrysum* plants. Furthermore, our findings point to the prioritization of candidate compounds that hold the greatest promise for further investigation in anti-cancer research.

## 4. Conclusions

The computational metabolomics study reported herein provides, for the first time, a global metabolic chart of the *Helichrysum splendidum* plant. The latter is characterized by a wide spectrum of chemical (sub)classes, which could be grouped into superclasses ranging from lipid and lipid-like molecules to organic oxygen compounds. The predominant chemical superclasses in the *H. splendidum* metabolomic landscape are (i) lipids and lipid-like molecules and (ii) phenylpropanoids and polyketides. This report on the metabolome of *H. splendidum* is the first of its kind, providing actionable insights on the chemical space of this plant. Further studies could include a comparative interrogation of the metabolome of this plant with the metabolic profiles of other Helichrysum species. Furthermore, computational methods were employed to predict potential anti-cancer compounds from *H. splendidum* metabolomic space. Network pharmacology points to flavonoid and terpenoid compounds, particularly rutin, luteolin, isorhamnetin, and oleanolic acid, as potential anti-cancer agents. Molecular docking predictively simulated the interactions between these compounds and CDK2 and CCNB1 proteins involved in cancer pathways and progression. The reported docking scores and molecular interactions indicate that *H. splendidum* extracts exhibit promising inhibitory activity against CDK2 and CCNB1 proteins. Thus, leveraging emerging computational and bioinformatics strategies, this study generates a metabolomic chart that describes the chemical space of *H. splendidum*, pointing to its potential anti-cancer candidates (metabolites and structurally related unknowns) from this plant. These actionable insights are a necessary step for further investigations (such as in vitro cell-culture-based assays) into *H. splendidum* flavonoids and terpenoids for confirmatory and translational studies towards anti-cancer drug discovery and treatments. 

## Figures and Tables

**Table 1:** List of annotated metabolites from *H. splendidum* methanol extracts.

Compound Name	Adduct	Precursor *m/z*	Library *m/z*	Mass Diff (Da)	Chemical Formula	RT (min)	Fragment Ions
Caffeic acid hexoside	[M-H]^−^	341.087	341.087	9.16 × 10^−5^	C_15_H_18_O_9_	5.83	179, 135
4-*O*-Caffeoylquinic acid	[M-H]^−^	353.09	353.09	0.002289	C_16_H_18_O_9_	5.99	191, 179, 173, 135
Quinic acid	[M-H]^−^	191.056	191.055	0.000305176	C_7_H_12_O_6_	6.03	191, 173, 127, 111
1,4-Dicaffeoylquinic acid	[M-H]^−^	515.119	515.12	0.000610352	C_25_H_24_O_12_	6.21	353, 191, 179, 135
Oryzanol A	[M-H]^−^	602.9	601.1594	1.7406	C_40_H_58_O_4_	6.28	601, 439, 409
3-*O*-Feruloylquinic acid	[M-H]^−^	367.103	367.103	0.000305176	C_17_H_20_O_9_	6.54	191, 173, 135
Pinicolic acid	[M-H]^−^	453.137	453.1793	0.0424194	C_30_H_44_O_34_	6.69	453
1,3-Dicaffeoylquinic acid	[M-H]^−^	515.119	515.12	0.000915527	C_25_H_24_O_12_	6.92	353, 191, 179, 135
5-*O*-Caffeoylquinic acid	[M-H]^−^	353.09	353.0869	0.00198364	C_16_H_18_O_9_	7.01	191, 179, 173, 135
Vitamin P	[M-H]^−^	609.146	609.146	0.000183105	C_27_H_30_O_16_	7.28	609, 301, 300
Isoquercetin	[M-H]^−^	463.088	463.088	0.000396729	C_21_H_20_O_12_	7.31	463, 300, 271, 255
Quercetin-3-*O*-glucosyl-6′’-acetate	[M-H]^−^	505.08	505.098	0.00189209	C_25_H_22_O_13_	7.51	505, 301, 300, 271
Ranolazine	[M-H]^−^	429.26	429.21	0.05	C_24_H_33_N_3_O_4_	7.6	429, 249
Gibberellic acid 8	[M-H]^−^	363.145	363.145	0.000976562	C_19_H_22_O_6_	7.71	363,301, 275
Kaempferol-3-*O*-rutinoside	[M-H]^−^	593.15	593.15	0.000915527	C_27_H_30_O_15_	7.72	593, 285, 284, 257
Kaempferol 3-glucuronide	[M-H]^−^	461.072	461.073	0.000183105	C_21_H_18_O_12_	7.76	285, 257
Isorhamnetin-3-*O*-glucoside	[M-H]^−^	477.104	477.104	9.16 × 10^−5^	C_22_H_22_O_12_	7.82	314, 299, 285, 271, 243
Viscidulin III	[M-H]^−^	345.06	345.0609	0.00109863	C_17_H_14_O_7_	8.68	330, 315, 287
Isorhamnetin	[M-H]^−^	315.05	315.05	0.000305176	C_16_H_12_O_7_	8.71	300, 271, 255
Eleutheroside E	[M-H]^−^	741.239	741.261	0.0216675	C_34_H_46_O_18_	8.71	741, 579, 417
Dihydroquercetin	[M-H]^−^	303.051	303.051	0.000396729	C_15_H_12_O_7_	8.84	175,125
Quercetin 3,7-dimethyl ether	[M-H]^−^	329.062	329.066	0.00479126	C_17_H_14_O_7_	9.43	314, 299, 271, 243
Jaceidin	[M-H]^−^	359.077	359.077	0.000305176	C_18_H_16_O_8_	9.49	344, 329, 314, 26
Cynarine	[M-H]^−^	515.12	515.12	0.00012207	C_25_H_24_O_12_	9.72	353, 191, 179
Pinocembrine	[M-H]^−^	255.066	255.066	0.000198364	C_15_H_12_O_4_	9.74	255, 213, 171, 151
Quercetin 3-*O*-glucuronide	[M-H]^−^	477.067	477.067	0.000823975	C_21_H_18_O_13_	9.86	301, 179, 151
Betulin	[M-H]^−^	442.7	441.1222	1.5778	C_30_H_50_O_2_	9.87	441, 260, 245, 231
Silybin	[M-H]^−^	482.4	481.113	1.287	C_25_H_22_O_10_	10.01	327, 315, 312
5-hydroxy-2-(4-hydroxy-3-methoxyphenyl)-3,6,7-trimethoxy-4H-chromen-4-one	[M-H]^−^	373.093	373.093	0.00088501	C_19_H_18_O_7_	10.15	358, 343, 328
Quercetin 3-*O*-malonylglucoside	[M-H]^−^	549.089	549.089	0.000427246	C_24_H_22_O_15_	10.16	300, 271,255
Kaempferol-3-*O*-glucoside	[M-H]^−^	447.094	447.093	0.000427246	C_21_H_19_O_11_	10.56	285, 284, 255, 227
3,7-Dihydroxy-3′,4′-dimethoxyflavone	[M-H]^−^	313.072	313.071	0.000183105	C_17_H_14_O_6_	10.59	298, 283, 255
Spiraeoside	[M-H]^−^	463.089	463.088	0.000488281	C_21_H_20_O_12_	10.6	301, 179, 151
Velutin	[M-H]^−^	313.072	313.071	0.000976562	C_17_H_14_O_6_	10.7	298, 283, 255
Kaempferol-3-*O*-glucuronoside	[M-H]^−^	461.073	461.073	0.00088501	C_21_H_18_O_12_	10.7	285, 257, 229
Tiliroside	[M-H]^−^	593.151	593.116	0.015625	C_30_H_26_O_13_	10.79	593, 285, 255
Rhein	[M-H]^−^	283.061	283.025	0.0357056	C_15_H_8_O_6_	11.31	268, 239, 211
9-hydroxy-10,12-octadecadienoic acid	[M-H]^−^	295.228	295.227	0.00241089	C_16_H_32_O_3_	11.91	295, 277, 195
3,4-di-*O*-caffeoylquinic acid	[M-H]^−^	515.12	515.12	0.000183105	C_25_H_24_O_12_	11.95	191, 179, 173
Luteolin	[M-H]^−^	285.04	285.04	0	C_15_H_10_O_6_	12.51	175, 151, 133
Limocitrin	[M-H]^−^	345.062	345.062	0.000396729	C_17_H_14_O_8_	12.64	315, 287
Dodecylbenzenesulfonic acid	[M-H]^−^	325.184	325.184	0.000183105	C_18_H_30_O_3_S	12.86	325, 183
Isokaempferide	[M-H]^−^	299.056	299.056	0.000213623	C_16_H_12_O_6_	13.98	255, 227
4′,5,7-Trihydroxy-3,6-dimethoxyflavone	[M-H]^−^	329.067	329.067	0.00088501	C_17_H_14_O_7_	13.99	299, 271, 215
Irigenin	[M-H]^−^	359.077	359.077	0.000305176	C_18_H_16_O_8_	14.62	329, 314,286, 258
3-*O*-Acetylpinobanksin	[M-H]^−^	313.066	313.071	0.000183105	C_17_H_14_O_6_	15.13	253
Tricin	[M-H]^−^	329.067	329.067	0.000396729	C_17_H_14_O_7_	15.3	314, 299, 271
Myricetin 3,7,3′,4′-tetramethyl ether	[M-H]^−^	373.093	373.093	0.000183105	C_19_H_18_O_8_	15.67	343, 328, 300, 285, 257
Eupatilin	[M-H]^−^	343.08	343.082	0.000305176	C_18_H_16_O_7_	16.89	298, 285, 270, 242
Decylbenzenesulfonic acid	[M-H]^−^	297.142	297.153	0.0107117	C_16_H_26_O_3_S	19.05	297, 183
Thymol-beta-d-glucoside	[M-H]^−^	311.15	311.168	0.0540771	C_24_H_32_O_10_	20.03	311, 183
Corosolic acid	[M-H]^−^	471.348	471.348	0	C_30_H_48_O_4_	20.33	471, 407
Hydroquinidine	[M-H]^−^	325.192	325.184	0.0105286	C_20_H_26_N_2_O_2_	21	325, 183
Canrenone	[M-H]^−^	339.197	339.2	0.00271606	C_22_H_28_O_3_	22.19	339, 184, 183
Oleanolic acid	[M-H]^−^	455.353	455.3533	0.000305176	C_30_H_48_O_3_	22.96	455

## Data Availability

Spectral data are available on the GNPS platform at these (job) links: https://gnps.ucsd.edu/ProteoSAFe/status.jsp?task=7bd698ee7b3443ac9246527e1b0e31f3 (accessed/generated on 17 March 2023). https://gnps.ucsd.edu/ProteoSAFe/status.jsp?task=132f6ff5c7494ffc9e7faace4874381d (accessed/generated on 17 March 2023).

## Supplementary Materials

The following supporting information can be downloaded at: https://www.mdpi.com/article/10.3390/metabo13101104/s1, Figure S1: LC-MS analyses of methanol extracts from *H splendidum*; Figure S2: Feature-based molecular network of *Helichrysum splendidum*; Table S1: Docking scores and glide energy of *Helichrysum splendidum* compounds with CNNB1 and CDK2 targets.
