# Supplementary material for: An Integrated Molecular Networking and Docking Approach to Characterize the Metabolome of Helichrysum splendidum and Its Pharmaceutical Potentials
Source: Metabolites. 2023 Oct 23;13(10):1104. doi: 10.3390/metabo13101104 (PMC10609414; doi:10.3390/metabo13101104)
Supplement: Supplementary file 1 [file metabolites-13-01104-s001.zip › metabolites-2635993-supplementary.pdf]

Supplementary Materials

An integrated molecular networking and docking approach to characterize the metabolome of *Helichrysum splendidum* and its pharmaceutical potentials

Motseoa Lephatsi[1], Mpho Choene[1], Abidemi Paul Kappo[1], Ntakadzeni E. Madala[2], Fidele Tugizimana[1,3]*

*** Correspondence:**
Corresponding Author, FT: ftugizimana@uj.ac.za   and Fidele.Tugizimana@omnia.co.za

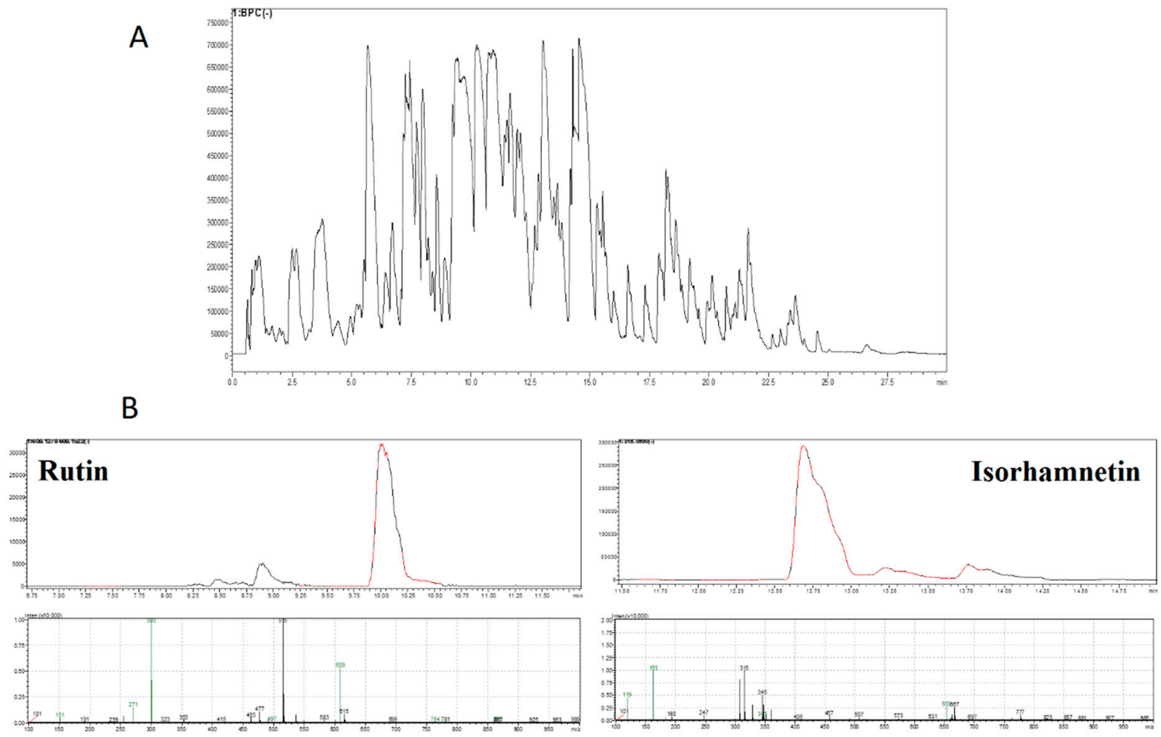

Supplementary Figure 1: LC-MS analyses of methanol extracts from *H splendidum*. (A) A typical mass chromatogram of the extracts, infographically displaying the complexity of the extracts in terms of peak population and intensities, which point to a complex mixture of metabolites with wide range of polarities. **(B)** Examples of annotated metabolites and their spectra.

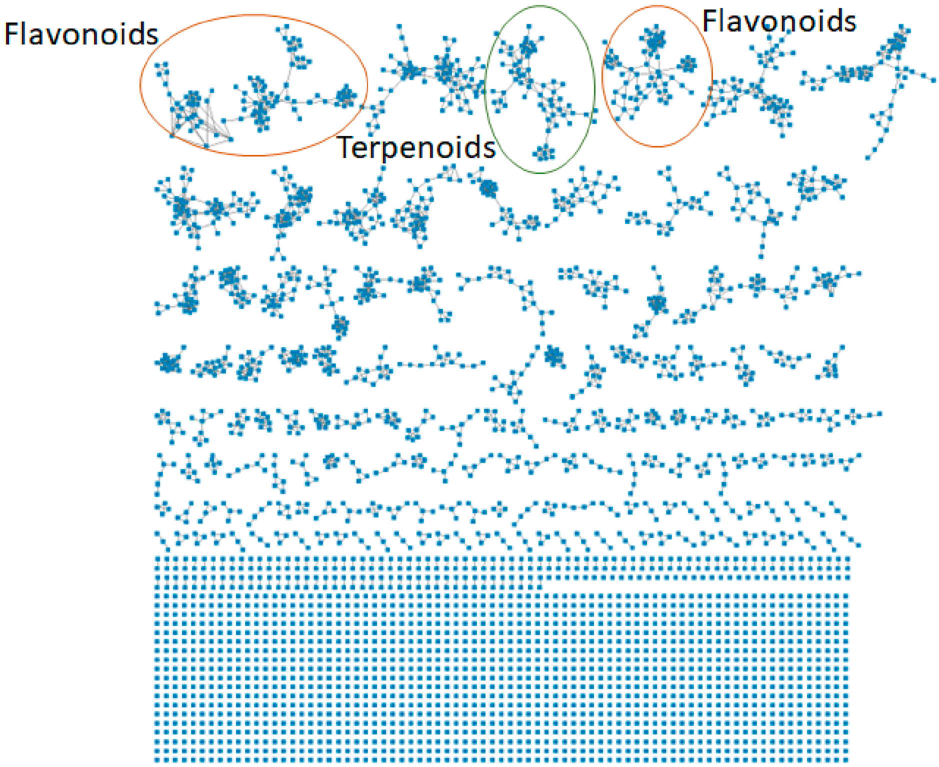

Supplementary Figure 2: Feature-based Molecular Network of *Helichrysum splendidum*. The nodes in the network represent different chemical features, while the edges represent the similarities between the features. The figure highlights different metabolites classes that were annotated, such as flavonoids and terpenoids.

Supplementary Table 1: Docking scores and glide energy of *Helichrysum splendidum* compounds with CNNB1 and CDK2 targets

Compound	Binding Affinity - CCNB1	Binding Affinity - CDK2	rmsd/ub	rmsd/lb
**Oleanolic acid**	-8.9	-8.9	0	0
**Luteolin**	-7.2	-7.3	0	0
**Rutin**	-7.5	-7.5	0	0
**Isorhamnetin**	-7.8	-8.9	0	0
